# Army Nurse Corps Coronavirus Disease (COVID-19) Lessons Learned

**DOI:** 10.1093/milmed/usab244

**Published:** 2021-09-01

**Authors:** Jack M Davis

**Affiliations:** Regional Health Command Pacific, Joint-Base Lewis–McChord, WA 98433, USA; U.S. Army Nurse Corps, Falls Church, VA 22042, USA

## Abstract

A new strain of coronavirus (COVID-19) emerged in 2020 changing the way the nation looked, worked and lived. In response to this unprecedented COVID-19 pandemic, the Army Nurse Corps (ANC) reexamined our capabies and agility to respond to a new and rapidly evolving environment. Maintaining the pivot to readiness, providing sustainable support and protecting our most valuable asset-our people-were and continues to be in the forefront of leaders’ thoughts as we faced this invisible adversary. With every new challenge, lessons learned provide an opportunity to re-examine challenges and successes of the response to COVID-19. Organizational restructuring, balancing risks, expanding capabilities and educational platforms were reassessed and adjusted to fill the needs of the environment as they evolved. The year 2020 will stand throughout history as another example where our readiness, resilience, and flexibility as an Army Nurse Corps was tried and tested. We demonstrated our ability to adapt and overcome-displaying our willingness to stand up as part of the Army Medicine Team and face an unknown adversary to protect the nation we vowed to serve.

The ANC supported the DoD, U.S. Army, and Army Medicine as it executed a multifaceted response to the COVID-19 pandemic (Fig. 1). Active and Reserve components supported a novel Urban Augmentation Medical Task Force (UAMTF) to deploy across the nation. UAMTF rapidly erected alternate care facilities and provided direct civilian hospital support for U.S. communities with the greatest need of additional resources and nursing care. Simultaneously, individual states and territories activated their National Guard to assist high-risk communities to protect their most vulnerable populations (**Fig. S1**). Army nurses stationed at military treatment facilities (MTFs) implemented a cross-cover plan to utilize nursing staff across multiple units. Additionally, Army nurses provided cross-coverage nursing care at Air Force and Navy MTFs within Defense Health Agency (DHA) Enhanced Multiservice Markets. Behavioral health teams, Combat Operations Stress Control teams, and chaplains were activated around the world to build resiliency, maintain the mental health and welfare of our Soldiers, and address potential for burnout across the force. ANC officers also supported Operation Warp Speed, the wide-scale national operation to produce and deliver safe and effective new COVID-19 vaccines in record time.

**FIGURE 1.**
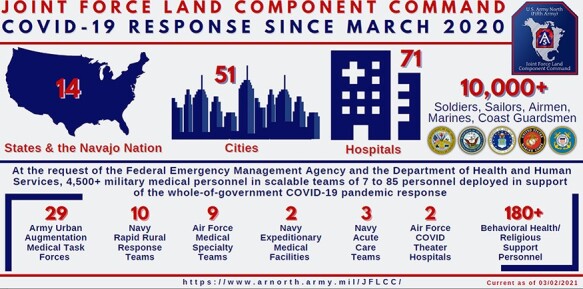
Joint Force Land Component Command (JFLCC) infograph.

Ongoing since March 26, 2020, U.S. Army North as U.S. Northern Command’s Joint Force Land Component Command has deployed over 4,500 military medical personnel to 71 hospitals, in 51 cities, in 14 states and the Navajo Nation, at the request of the Federal Emergency Management Agency and the Department of Health and Human Services in support of the whole-of-America response to the COVID-19 pandemic (U.S. Army graphic by Lauren Padden/U.S. Army North Public Affairs)^2^.

Our public health team’s unique knowledge base, training, and talents were vital to our ability to implement a rapid response to the COVID-19 pandemic. Army public health nurses served as community leaders and installation advisors (Fig. 2), providing evidence-based recommendations to assist garrison commanders in determining Health Protection Condition (HPCON) levels and installation response. These public health nurses rapidly expanded contact tracing teams (**Fig. S2**) for surveillance and disease containment interventions, deployed with each of the field hospitals to support civilian communities and across the globe to places such as South Korea, Guam, and U.S. Naval Ship Theodore Roosevelt (Fig. 3).

**FIGURE 2.**
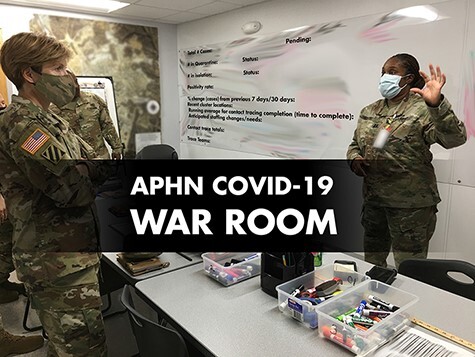
Army public health war room.

Capt. Celeste Singletary, Army Public Health Nurse, briefs Brigadier General Paula Lodi, Regional Health Command – Atlantic Commanding General – regarding Public Health Nursing's efforts to track and provide up-to-date installation level COVID-19 information to improve commanders' abilities to make informed decisions. The Public Health team worked to identify cluster cases, upward and downward trends, positivity rates, and conducted unit trainings for Team Clean, Team Trace, and Contact Tracing (Official U.S. Army photo).

**FIGURE 3.**
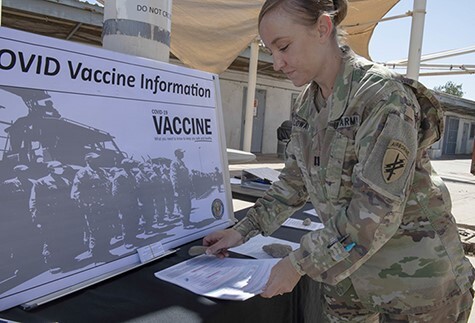
Camp Lemonnier, Djibouti professionals’ host vaccine information booth.

U.S. Army Capt. Shantyl Galloway of Fort Knox, Ky., assigned to the 443rd Civil Affairs Battalion, sets up a COVID vaccine information booth on Camp Lemonnier, Djibouti, February 2, 2021. The informational booth is part of a regional campaign to help educate military members about the voluntary COVID vaccination. (U.S. Navy photo by Mass Communication Specialist 1st Class Natalia Murillo)^3^.

Army forces participated in numerous Defense Support to Civil Authorities (DSCA) missions across the nation serving as a support element within the national response framework.

This critical support was achieved by working with interagency partners including the Federal Emergency Management Agency and local and state government agencies (Table I). The utilization of UAMTF deployed in support of these efforts. In Seattle, Washington, UAMTF-627 under Task Force 46 West set up a 250-bed hospital in CenturyLink Field from March to April 2020, providing full-spectrum Role 3 medical care hospitalization. From July to September 2020 under Task Force 51, UAMTF-627 deployed again to San Antonio, Texas, to provide Health Service Support (HSS) to COVD-19 patients by embedding these teams into five civilian hospitals. In those three months, staff worked 2,288 shifts and provided over 25,682 hours of HSS^1^. According to the 44th Medical Brigade Chief Nursing Officer, Colonel D. Phillips, Army nurses helped comprise Joint Task Force Silver Dragons—a multicomponent, joint interagency force. This task force supported six northeastern states from Virginia to Maine with 12 UAMTFs embedding medical assets into 21 civilian hospitals, stood up seven alternate care facilities such as the Javits Center (**Fig. S3**), and supported the USNS Comfort (T-AH 20) in New York. Their support was crucial for the success of local response in major cities across the USA during a rapid onset of COVID-19 outbreaks.

**TABLE I.** U.S. Army North COVID-19 Hospital Support Since November 2020

State	Personnel	Locations
Arizona	Approximately 70 military medical personnel from U.S. Army Reserve and U.S. Air Force	Two hospitals in two cities: Kingman Regional Medical Center from March to April 2021 Yuma Regional Medical Center from January to February 2021
Navajo Nation (in Arizona and New Mexico)	Approximately 50 military medical personnel from the U.S. Army Reserve and U.S. Navy-supported DHHS, Indian Health Service, and Navajo Nation	Four hospitals in four cities: Tuba City Medical Center from January to March 2021 Chinle Comprehensive Health Care Facility from December to March 2021 North Navajo Medical Center from December to March 2021 Gallup Indian Medical Center from January to March 2021
Texas	Approximately 140 military medical personnel from U.S. Army Reserves, U.S. Navy, and U.S. Air Force	Six hospitals in four cities: Hendrick Medical Center from January to February 2021 CHI St. Luke’s Health-Memorial Hospital from January to February 2021 Fort Duncan Regional Medical Center from January to February 2021 Hospitals of Providence Transmountain Campus from November to December 2020 University Medical Center of El Paso from November to December 2020 Las Palmas Del Sol Medical Center from November to December 2020
California	Approximately 225 military medical personnel from U.S. Army and U.S. Air Force	Eight hospitals in seven cities: Arrowhead Regional Medical Center from December 2020 to January 2021 Dameron Hospital from December 2020 to January 2021 Harbor-UCLA Medical Center from January to February 2021 Adventist Health Lodi Memorial Hospital from December 2020 to February 2021 Adventist Health White Memorial from January to February 2021 Community Regional Medical Center from December 2020 to February 2021 LAC + USC Medical Center from December 2020 to February 2021 Riverside University Health System Medical Center from January to February 2021
Wisconsin	Approximately 45 military medical personnel from the U.S. Army	Four hospitals in four cities from December 2020 to January 2021: Marshfield Medical Center in Marshfield Marshfield Medical Center in Eau Claire Marshfield Medical Center in Beaver Dam Marshfield Medical Center in Rice Lake

U.S Army North, U.S. Northern Command’s Joint Force Land Component Command, has overseen the DoD’s COVID-19 response operations in support of the Federal Emergency Management Agency and the Department of Health and Human Services since March 2020. Approximately 590 military medical personnel from the U.S. Army and U.S. Army Reserve, the U.S. Navy, and the U.S. Air Force recently worked alongside civilian health care providers in civilian hospitals, helping threat COVID-19 patients in six states and the Navajo Nation as part of the whole-of-government response to the pandemic. (U.S. Army North Fact Sheet, 2021)^4^.

## ORGANIZATIONAL RESTRUCTURING

With every new challenge, there are lessons learned. At the onset of the pandemic, the unknowns about COVID-19 vastly outweighed the knowns of the disease. The necessity to respond expeditiously required preparations that had not yet been defined from both the individual services and the DHA. This resulted in many parallel lines of effort. Each service had command and control of their respective MTFs responsible for collaborating with their supported installations and other major military commands supporting the national response.

Reporting requirements was challenging during the initial phases of the pandemic response because of overlapping multiple lines of authority and communication. One solution implemented by the Medical Command (MEDCOM) team was ensuring that clear and concise messages were provided for status reporting, expansion, and degradation of capabilities, and managing expectations, projecting needs, and finding shared concept of operations became necessary to bring structure to a chaotic situation.

### Balancing Risks

**FIGURE 4.**
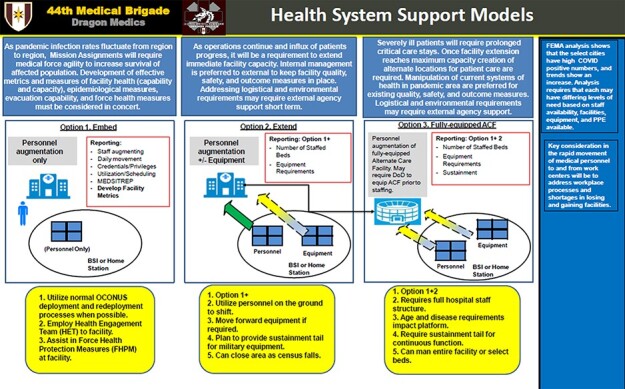
44th medical brigade health system support.

In the process of readjusting and resetting capabilities, leaders were tasked to balance the protection of people, residual risk reduction, and a high operational tempo. A consensus on levels of risk and acceptable risk had to be made for each unique organization. Time-sensitive decisions with minimal information demonstrated the use of prudent risk to the fullest extent. Leadership made challenging decisions on the supply chain, exploitation of nursing skill sets while maintaining quality and safety, and modification of available services under emergency situations. UAMTFs were rapidly deployed to high-risk locations with an initial plan that changed in the short distance from concept to implementation. As the missions and locations shifted, equipment and personnel support configurations shifted (Fig. 4). Embedding UAMTF personnel into civilian hospital facilities later became the preferred support response (**Fig. S4**), and retiree recalls were in effect because of nationwide staffing shortage. The scope of implementing large-scale contract tracing and surveillance within our public health teams quickly surpassed their organic capabilities. Expeditious expansion of contact tracers through volunteers was imperative to reduce transition in high-prevalence areas.

44th Medical Brigade Chief Nursing Officer outlined planning considerations required during Task Force 261 support to the northeastern section of the United States during the Portuguese 3rd International Meeting of Military Nursing on September 17, 2020. (U.S. Army graphic by Col. Douglas Phillips)^5^.

### Military Treatment Facility Patient Bed Expansion Plans

MTFs historically had bed expansion plans to accommodate a large influx of inpatients during an influenza pandemic. Upon activation for the COVID-19 pandemic, testing of those plans for operability identified gaps that were addressed. Nurse leaders began reworking on existing bed expansion plans, factoring in decreased staffing because of the potential loss of activated reserve nurses to support missions outside the MTF and loss of staff to COVID-19 infection. Hospitals erected field tents in parking lots, and drive-through testing sites were designed to provide external space for an influx of patients requiring screening and testing. Our supply chains were instantaneously strained with the increased need in personal protection equipment and the growing requirement for new laboratory testing capabilities.

### Operational Capabilities Re-Examined

Our operational unit structures were developed and designed for combat operations. We soon recognized that the skills and talents of the whole unit were not ideal in facing this new threat and would require adaptation. With COVID-19 conditions, traditional Role 3 operation force structure provided excess capacity in primary care, emergency medicine, and surgery while straining inpatient nursing assets with the provision of high acuity, prolonged care. Individual augmentees were utilized to fill professional shortages. Our Role 3 and Role 4 structures needed the flexibility to quickly reset and adjust to get the right mix of personnel to the response to this unique threat. The critical wartime areas of concentrations (AOCs) of critical care nurses, emergency nurses, and certified nurse anesthetists proved highest in demand. With the inventory shortages already felt in these specialties, the pandemic emphasized the imperative for all ANC officers to maintain clinical competence and cross-train to assist in a clinical response. By pulling necessary skills across the force to supplement the expanding needs within Role 3 and Role 4 units going to high-risk locations, we risked leaving local MTFs to sustain home installation health care operations with diminished numbers of staff. Alternative staffing plans were developed to include setting up skills training and education to allow ambulatory and administrative nursing staff, who were normally far away from the bedside, to have the flexibility and confidence to support inpatient and critical care environments should the need exceed current capacity.

### Adaptations to ANC Educational Programs

The ANC modified our education programs as instructors, and students were critically needed to support the COVID-19 response. The Clinical Nurse Transition Program temporarily modified from a 24-week program to a 14-week program with a primary focus on clinical training to assist with the growing demand for inpatient nurses at the bedside. Adjustments were made to alter required training and education, including AOCs producing courses, through reduced seating capacity, modified training capabilities, and leveraging of virtual learning opportunities. Medical Center of Excellence nursing courses, such as the Clinical Nurse Officer in Charge and Non-Commissioned Officer in Charge (CNOIC/NCOIC) Course and Entry-Level Nurse Executive Course (ELENC), transitioned to Microsoft Teams for fully virtual offerings, whereas other courses such as Sexual Assault Medical Forensic Examiner formatted to a hybrid approach. Students completed a 2-week restriction of movement: completed week 1 training virtually from their hotel room and finished week 2 hand-on practicum in class socially distanced or at facilities using trained preceptors. Although initially challenging to adapt courses into new delivery platforms in such a short time, course quality and information remained intact. These newly modified virtual courses provided a greater reach across the force while simultaneously continuing the training mission in a safe environment.

### ANCReadiness Indicators

From these lessons learned, the ANC is focusing on reassessing current structures and readiness indicators. One focus area is the Individual Critical Task List (ICTL). This is an Army Medicine initiative focused on identifying and measuring proficiency in the critical wartime skills required for deploying a Ready Medical Force. The ANC is actively re-engaging our ICTL tasks with the consultant for every AOC to review, revise, and reintroduce to the field, highlighting the critical need to maintain proficiency in our skills. The ICTL directly enables the operational unit’s proficiency to perform collective tasks. In turn, collective tasks enable the operational unit’s capability and readiness to execute its mission essential task list (METL) (**Fig. S5**). We are also relooking our organizational structures and doctrinal plans to ensure efficient and effective skill mix, expansion and restructure abilities, and position descriptions. As understanding and scientific knowledge of this rapidly evolving threat became clearer, our ability to fill needs of the environment as they evolved showcased the diverse talents and unique value of the ANC to Army Medicine.

The year 2020 will stand throughout history as another example where our readiness, resilience, and flexibility as an ANC were tried and tested. We demonstrated our ability to adapt and overcome, displaying our willingness to stand up as part of the Army Medicine Team and face an unknown adversary to protect the nation we vowed to serve. One thing was made clear through it all—we have outstanding leaders who courageously held the torch in the darkest of night and lit the way for others to follow. Anytime, Anywhere, and Always Ready.

## Supplementary Material

usab244_Supp
