# Supplementary material for: Army Nurse Corps Coronavirus Disease (COVID-19) Lessons Learned
Source: Mil Med. 2021 Sep 1;186(Suppl 2):4–8. doi: 10.1093/milmed/usab244 (PMC8499829; doi:10.1093/milmed/usab244)
Supplement: usab244_Supp [file usab244_supp.zip › Supplemental_Fig 1.pdf]

Supplemental 1: Maryland National Guard Gives Assistance to Nursing Homes

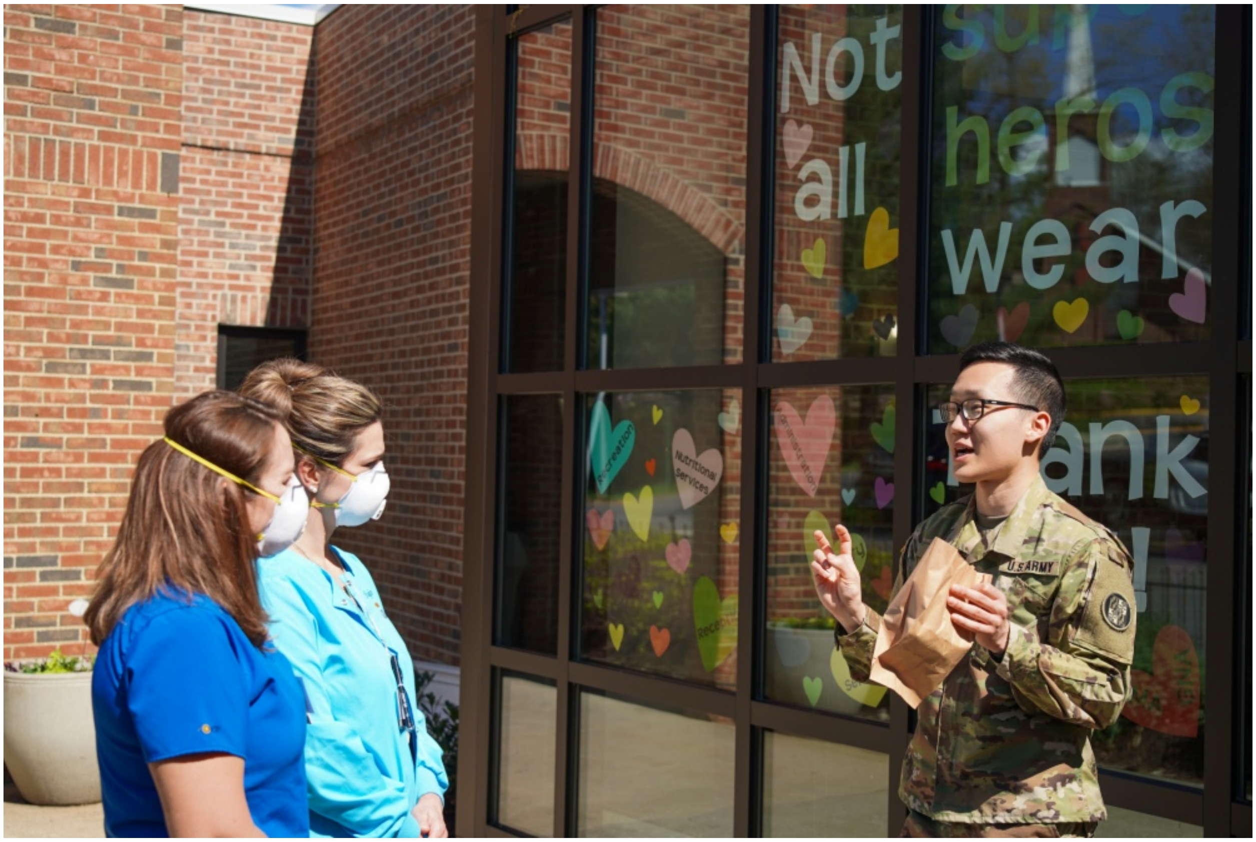

2nd Lt. Justin Lee, Medical Surgical Nurse with the 104th Medical Company Area Support, speaks with Celia Goughenour, Director of Nursing, and Mary Wheat, Director in Training, outside of Crofton Care & Rehabilitation Center in Crofton, Maryland, April 9, 2020. The Maryland National Guard is offering assistance to nursing homes across the state to ensure facilities have everything they need to combat the COVID-19 virus. (Photo by U.S. Army National Guard Sgt. James Nowell) Retrieved from https://www.dvidshub.net/image/6172034/maryland-national-guard-gives-assistance-nursing-homes. Accessed on May 07, 2021.
