# Supplementary material for: Army Nurse Corps Coronavirus Disease (COVID-19) Lessons Learned
Source: Mil Med. 2021 Sep 1;186(Suppl 2):4–8. doi: 10.1093/milmed/usab244 (PMC8499829; doi:10.1093/milmed/usab244)
Supplement: usab244_Supp [file usab244_supp.zip › Supplemental_Fig 2.pdf]

Supplemental 2: Army Public Health Nurse Never Misses an Opportunity to Give Back

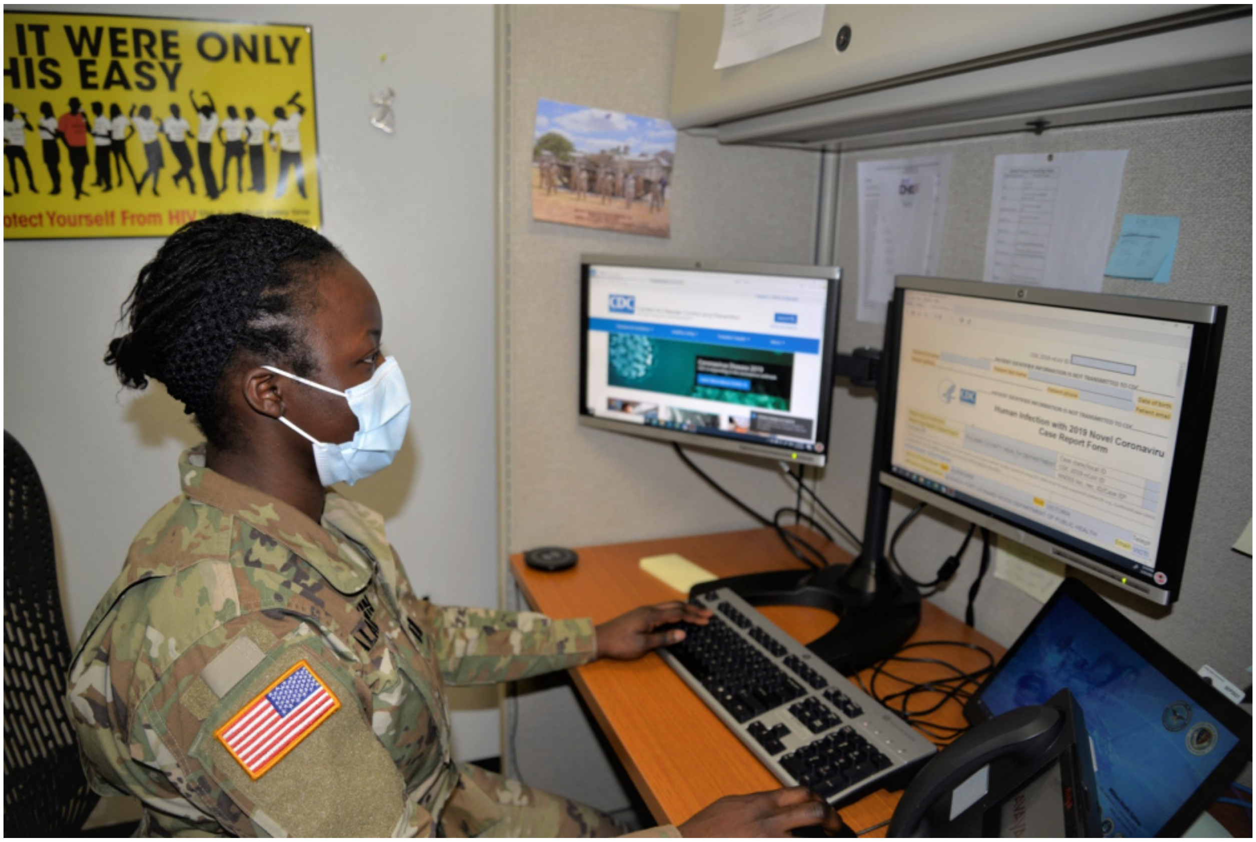

Capt. Victoria Aladeokin glances at a contact tracing form during her duties as an Army Public Health Nurse on Fort Leonard Wood, Missouri Feb. 25. (U.S. Army photo by Chad Ashe) Retrieved from https://www.dvidshub.net/image/6550162/army-public-health-nurse-never-misses-opportunity-give-back. Accessed on May 07, 2021
