# Supplementary material for: Army Nurse Corps Coronavirus Disease (COVID-19) Lessons Learned
Source: Mil Med. 2021 Sep 1;186(Suppl 2):4–8. doi: 10.1093/milmed/usab244 (PMC8499829; doi:10.1093/milmed/usab244)
Supplement: usab244_Supp [file usab244_supp.zip › Supplemental_Fig 3.pdf]

Supplemental 3: Nurse Anesthetist Mobilized to NYC to Assist in COVID-19 Response

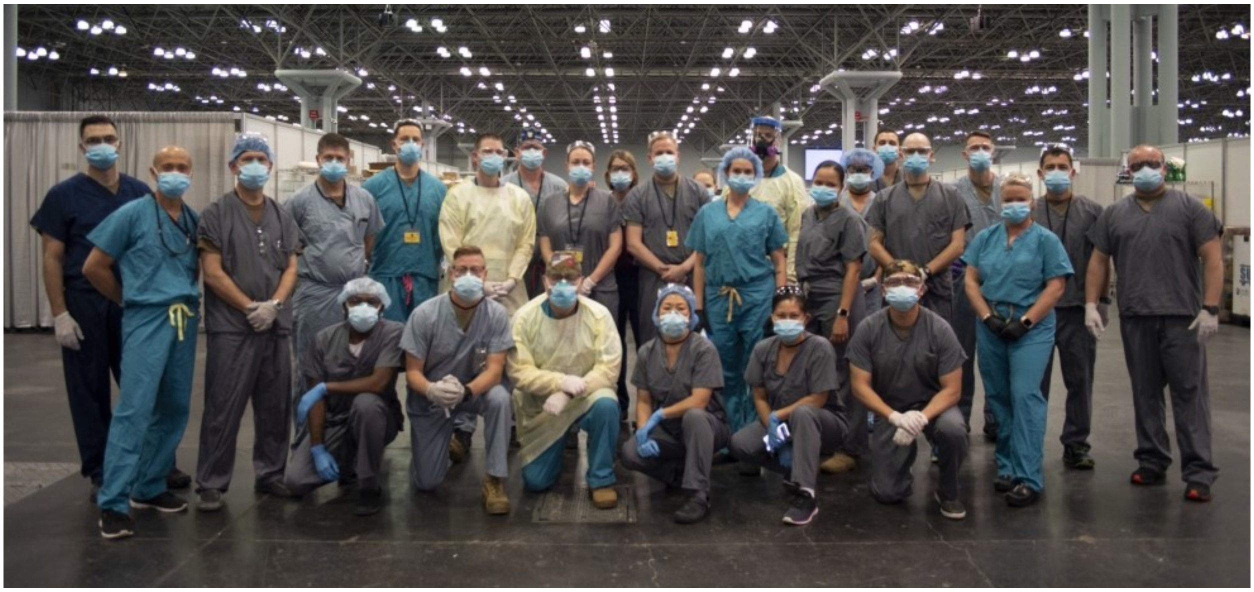

U.S. Army Lt. Col. Aaron Gopp (far left), and 26 fellow nurse anesthetists at the Javits Center, New York City, Apr 27, 2020. The military medical professionals were part of the Department of Defense response to COVID-19. (Official U.S. Army photo) Retrieved from https://www.dvidshub.net/image/6206822/nurse-anesthetist-mobilized-nyc-assist-covid-19-response. Accessed on May 07, 2021.
