# Supplementary material for: Army Nurse Corps Coronavirus Disease (COVID-19) Lessons Learned
Source: Mil Med. 2021 Sep 1;186(Suppl 2):4–8. doi: 10.1093/milmed/usab244 (PMC8499829; doi:10.1093/milmed/usab244)
Supplement: usab244_Supp [file usab244_supp.zip › Supplemental_Fig 4.pdf]

Supplemental 4: Army Nurses Serve in California Hospital

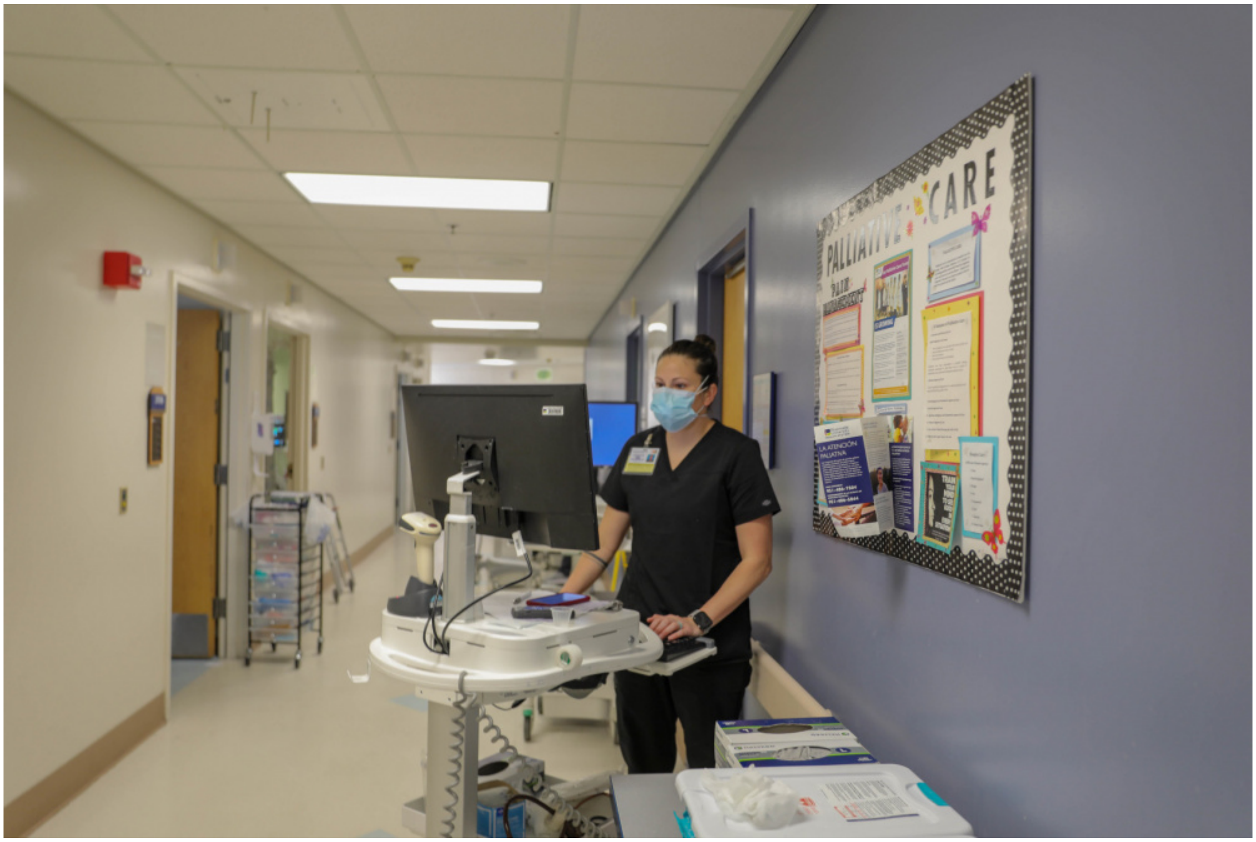

U.S. Army Maj. Tori Salas, assigned to the 627th Hospital Center, Fort Carson, Colo., charts her observations of her patients at the Riverside University Healthcare System, Riverside, Calif., Jan. 11, 2021. Salas works alongside other registered nurses in the step-down unit where an intermediate level of care is provided between the intensive care units and the general medical-surgical wards. U.S. Northern Command, through U.S. Army North, remains committed to providing flexible Department of Defense support to the whole-of-America COVID-19 response. (U.S. Army photo by Spc. Preston Robinson/5th Mobile Public Affairs Detachment) Retrieved from https://www.dvidshub.net/image/6479190/army-nurses-serve-california-hospital. Accessed on May 07, 2021.
