# Supplementary material for: Army Nurse Corps Coronavirus Disease (COVID-19) Lessons Learned
Source: Mil Med. 2021 Sep 1;186(Suppl 2):4–8. doi: 10.1093/milmed/usab244 (PMC8499829; doi:10.1093/milmed/usab244)
Supplement: usab244_Supp [file usab244_supp.zip › Supplemental_Fig 5.pdf]

Supplemental 5: U.S. Army Reserve Nurses Support COVID-19 Fight in Yuma

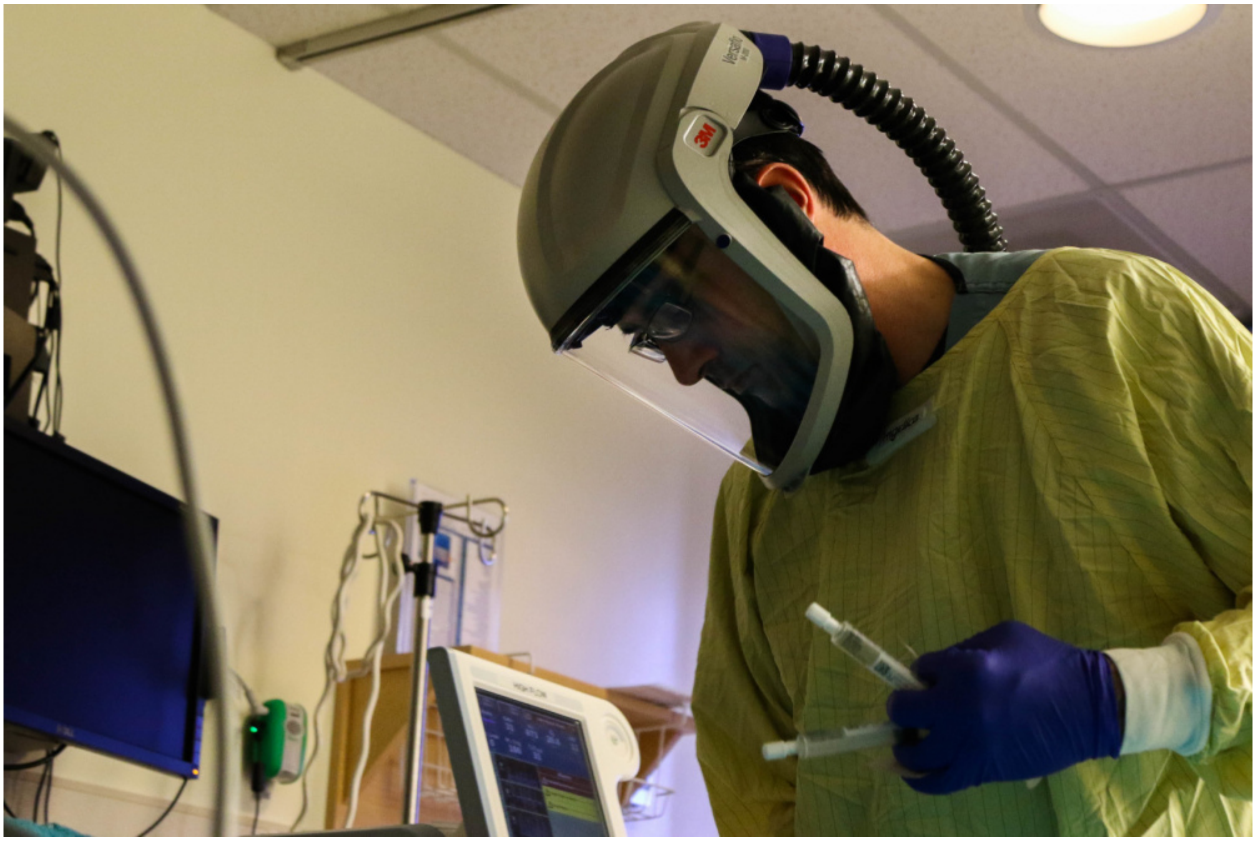

U.S. Army Maj. Jon Cuzner, a registered nurse with U.S. Army Reserve Urban Augmentation Medical Task Force 328-1, assigned to support Yuma Regional Medical Center, Yuma, Ariz., flushes an IV for one of his patients in the hospital's Medical/Surgical ward where COVID-19 patients are cared for, Jan. 15, 2021. The IV flush helps Cuzner ensure that fluids are flowing correctly to the patient he cares for while supporting the hospital's fight against COVID-19 as an integrated part of the medical staff. U.S. Northern Command, through U.S. Army North, remains committed to providing flexible Department of Defense support to the whole-of-America COVID-19 response. (U.S. Army photo by Maj. Doug Halleaux) Retrieved from https://www.dvidshub.net/image/6484071/us-army-reserve-nurses-support-covid-19-fight-yuma. Accessed on May 07, 2021.
